# Urban Adolescents’ Physical Activity Experience, Physical Activity Levels, and Use of Screen-Based Media during Leisure Time: A Structural Model

**DOI:** 10.3389/fpsyg.2017.02317

**Published:** 2018-01-23

**Authors:** Hui Xie, Jason L. Scott, Linda L. Caldwell

**Affiliations:** ^1^Department of Recreation and Tourism Management, California State University, Northridge, Northridge, CA, United States; ^2^Department of Kinesiology, Recreation, and Sport Studies, The University of Tennessee, Knoxville, TN, United States; ^3^Department of Recreation, Park and Tourism Management, The Pennsylvania State University, University Park, PA, United States

**Keywords:** sedentary behavior, screen-based media, physical activity, leisure, adolescents

## Abstract

There is limited understanding of the relationship between physical activity and use of screen-based media, two important behaviors associated with adolescents’ health outcomes. To understand this relationship, researchers may need to consider not only physical activity level but also physical activity experience (i.e., affective experience obtained from doing physical activity). Using a sample predominantly consisting of African and Latino American urban adolescents, this study examined the interrelationships between physical activity experience, physical activity level, and use of screen-based media during leisure time. Data collected using self-report, paper and pencil surveys was analyzed using structural equation modeling. Results showed that physical activity experience was positively associated with physical activity level and had a direct negative relationship with use of non-active video games for males and a direct negative relationship with use of computer/Internet for both genders, after controlling for physical activity level. Physical activity level did not have a direct relationship with use of non-active video games or computer/Internet. However, physical activity level had a direct negative association with use of TV/movies. This study suggests that physical activity experience may play an important role in promoting physical activity and thwarting use of screen-based media among adolescents.

## Introduction

U.S. adolescents have a high incidence of physical inactivity and spend a large portion of their free time using screen-based media such as TV, cellphones, and video games (Strasburger et al., 2010, 2012; Kann et al., 2016; National Physical Activity Plan Alliance, 2016), which may contribute to various health problems. According to the most up-to-date U.S. physical activity data measured by accelerometers (2005–2006 NHANES), only 7.5% youth between the ages of 12–15 and 5.1% youth between the ages of 16–19 meet the U.S. physical activity guidelines (Katzmarzyk et al., 2016). In the meantime, more than two thirds of youth (ages 12–19) fail to meet the screen-time recommendation by American Academy of Pediatrics (National Physical Activity Plan Alliance, 2016). This is particularly true for Latino and African American adolescents, many of whom are from families of low socioeconomic status. For example, Latino and African American students of 9th through 12th grade have a lower rate of meeting the U.S. physical activity guideline than White students (Kann et al., 2016). In addition, Latino and African American adolescents on average spend more time on screen-based media such as TV and videogames than their White counterparts (Rideout et al., 2010; Murphey et al., 2014; Common Sense Media, 2015).

To prevent these potential negative health consequences, one needs to be concerned with adolescents’ physical activity (PA) as well as their unhealthy use of screen-based media (Kruger et al., 2007). However, there is no guarantee that increased physical activity will lead to decreased sedentary activity, like screen-based media use. Recent studies found these two types of behaviors may co-exist among certain adolescents (Liu et al., 2010; Taverno Ross et al., 2016). This is not surprising because adolescents normally have the freedom and enough time to do both types of activities. Furthermore, if adolescents do not enjoy physical activity, but participate due to internal or external pressure (i.e., controlled self-regulation; Moller et al., 2006; Baumeister et al., 2007b), they may deplete their willpower, and as a result, become even less likely to refrain from doing certain sedentary activities, such as playing video games (Schmeichel and Baumeister, 2004). This implicates the role of positive physical activity experience in promoting adolescents’ physical activity and thwarting unhealthy sedentary activities, which has not been vigorously studied. Furthermore, the study of physical activity experience may be particularly important to Latino/African American adolescents living in disadvantaged communities as it is usually more difficult for them to obtain positive physical activity experience due to lack of supportive physical and social environment (e.g., lack of physical activity programs and facilities, insufficient parental support, maintenance and safety issues at physical activity locations; Duke et al., 2003; Dahmann et al., 2010). Thus, this study aims to examine the interrelationships between leisure time physical activity experience (PA-E), leisure time physical activity levels (PA-L), and leisure time use of different types of screen-based media (UM; i.e., TV/movies, computer/Internet, and non-active video games), using a sample predominantly comprised of Latino and African American urban adolescents.

## Physical Activity Experience, Physical Activity Levels, and Use of Screen-Based Media

Affective experience is powerful in guiding human behavior, and in many situations, has a stronger impact on individuals’ behavior than cognition (Edwards, 1990; Breckler and Wiggins, 1991; Lavine et al., 1998). This is particularly true for adolescents. Recent studies in neuroscience found that compared to adults, adolescent decision making/behavior was more influenced by their affective state (Giedd et al., 1999; Yurgelun-Todd and Killgore, 2006; Casey et al., 2008).

In the area of physical activity and sports, positive affective experience, such as physical activity enjoyment, has been found to be positively associated with physical activity participation among children and adolescents (DiLorenzo et al., 1998; Sallis et al., 1999; Dishman et al., 2005). In addition, the positive experience derived from physical activities may include other affective states, such as feeling of pride. Therefore, in this study we included multiple positive leisure time physical activity experiences such as enjoyment, sense of accomplishment, and feeling of being absorbed (Csikszentmihalyi, 1990; Caldwell et al., 1992; Mannell and Kleiber, 1997; Moore et al., 2009). In line with the above-mentioned studies, we posit that positive physical activity experience will increase adolescents’ physical activity levels during leisure time.

When adolescents participate in leisure time physical activity, they may spend less time on different types of screen-based media. However, the relationship between the two may be complicated by a number of factors. First, adolescents usually have sufficient amounts of leisure time, and because of that, increasing leisure time physical activity does not necessarily require adolescents to reduce their use of screen-based media. If adolescents do not obtain positive experiences from physical activity, they may seek desirable experiences from screen-based media such as video games or TV. Second, according to self-regulation theory (Baumeister et al., 2007a), when adolescents engage in physical activity due to the feeling of external pressures (e.g., playing sports because of parents’ requirement) that contradicts their values, beliefs, or desire (i.e., controlled self-regulation; Moller et al., 2006; Baumeister et al., 2007b), they may deplete their willpower and become temporarily less capable of resisting the subsequent temptation from certain screen-based media (Schmeichel and Baumeister, 2004). Third, if adolescents strive to finish their physical activity goals or requirements, they may feel more justified or deserving to use the screen-based media later, a phenomenon called “licensing effect” (Merritt et al., 2010). These theories seem to suggest that although leisure time physical activity participation may to some extent reduce adolescents’ leisure time available for screen-based media, leisure time physical activity experience seems to play an independent, and probably a more critical role in pulling adolescents out of the screen-based media.

In summary, the purpose of this study is to investigate the interrelationship between leisure time physical activity experience (PA-E), leisure time physical activity level (PA-L), and leisure time use of screen-based media (UM) including TV/movies, computer/Internet, and non-active video games. We posit that PA-E will have a positive effect on PA-L, which in turn will affect different types of UM. In addition, we expect that PA-E will have a direct negative effect on different types of UM. Furthermore, we explored potential gender differences in these relationships since male and female adolescents may differ in socialization (Hill and Lynch, 1983; Priess et al., 2009) and affect/emotional regulation and internalization (Chaplin and Aldao, 2013).

## Materials and Methods

### Sample and Data

Data for this study come from a larger study aimed at developing self-report physical activity measures and assessing attitudes, motivations and values adolescents have about physical activity. The study sample consists of 305 students (46.8% female) from 7th and 8th grades in four schools located in an urban, under-resourced area within a city in the Northeast United States. Ninety to ninety-five percent of students in the participating schools were African Americans or Latino Americans, with approximately 80% of the students being eligible for free or reduced price lunch. The students were recruited to participate in the study during homeroom classes. Data were collected using self-report, paper and pencil surveys. Surveys were administered during scheduled homeroom hours in Spring 2010. The study was approved by the institutional review board of the Pennsylvania State University. Opt-out consent was obtained from students’ parents. Parents provided written affirmation if they did not want their children to participate in the study. Only students who had parental consent and provided written assent participated in the study.

### Measures

#### Leisure Time Physical Activity Experience (PA-E)

Physical activity experience was conceptualized based on previous research on positive adolescent leisure experience and physical activity enjoyment (Csikszentmihalyi, 1990; Caldwell et al., 1992; Mannell and Kleiber, 1997; Moore et al., 2009). The construct consists of five items, which address enjoyment, sense of accomplishment, feeling of pride, and absorption. Sample items read: “*When you are doing physically active things in your free time, how often do you enjoy it/does it give you a sense of accomplishment?*” (1 = Never; 5 = All the time).

#### Leisure Time Physical Activity Level (PA-L)

Physical activity level was assessed using self-report measures that were developed based on a review of existing physical activity literature (Sallis and Saelens, 2000; Treuth et al., 2003, 2005; Wong et al., 2006; Welk et al., 2007). The survey questions shared similar contents and format with Physical Activity Questionnaire for Adolescents (PAQ-A; Kowalski et al., 1997) and were specifically modified to gather physical activity information from minority adolescents in an urban environment. Participants were asked to recall the amount of time they usually spent on walking, running, and other physical activity in the last 7 days during six leisure time periods, including two periods during school days: (1) after school (until 6 pm), (2) evenings of school days (after 6 pm); and four periods during weekend: (3) Saturday morning, (4) Saturday afternoon and evening, (5) Sunday morning, and (6) Sunday afternoon and evening. Each question addressed *one* type of physical activity during *one* period.

Walking and running during school days was assessed using the following questions: *“During the past 5 school days, how much time [SPECIFIC PERIOD], did you usually spend [SPECIFIC PA] each day?”* To assess weekend walking and running, participants were asked *“How much time [SPECIFIC PERIOD], did you spend [SPECIFIC PA]?”* (1 = “0 min” to 7 = “60 min or more,” in increments of 10 min).

Other physical activity during school days was evaluated using the following procedure. First, participants were asked “*During the past 5 school days, if you did any of the following physical activities [SPECIFIC PERIOD], circle the ONE activity you did most.”* The response list include of 13 activities such as baseball, cheerleading, dancing, riding a bike, and lifting weights, in addition to “other” and “I did not do any of these.” Then, participants were asked “*How many days did you do this activity [SPECIFIC PERIOD]?*” (1 = “0 day” to 6 = “5 days”). For weekend, the same question was used to identify participants’ most frequent physical activity during each period. Then participants were asked to indicate the amount of time they spent on the activity during each period (1 = “0 min” to 7 = “60 min or more,” in increments of 10 min). The scores of two items measured on the six-point scale were converted to the seven-point scale as the rest of the items using Little’s (2013, p. 19) procedure.

It should be noted that our goal was not to estimate total physical activity time for the participants, but to use three types of activities during different times of periods as indicators of how physically active participants were (i.e., physical activity levels). To make a more concise representation of each type of physical activity, we averaged the scores for (1) afterschool period and evening during school days, (2) Saturday morning and Saturday afternoon/evening, and (3) Sunday morning and Sunday afternoon/evening. As a result, PA-L was measured by nine items: walking, jogging, and other physical activity during three periods: afterschool, Saturday, and Sunday.

#### Leisure Time Use of Screen-Based Media (UM)

Participants were asked to recall the amount of time they usually spent per day on watching TV/movies, using computer/Internet, and playing non-active video games in the last 7 days during two leisure time periods: (1) after school and evenings of school days; and (2) Saturday and Sunday. Six questions were used with each question addressing *one* type of screen use during *one* period. UM during school days were measured using the following questions: “*During the past 5 school days, how much time did you usually spend [SPECIFIC MEDIUM USE] after school (and evening) each day?”* (1 = “None” to 8 = “4 h and more”) UM during the past weekend were measured using the following questions: *“How much time over both days did you spend [SPECIFIC MEDIUM USE] on Saturday and Sunday?”* (1 = “None” to 10 = “7 h and more”) The scores on the 10-point scale were converted to the 8-point scale as the rest of the items using Little’s (2013, p. 19) procedure. Then, a composite score was created respectively for each type of media by averaging the measures for the two periods.

### Data Analysis

#### Parceling

Parceling refers to pairing and aggregating individual items of a latent construct to form a smaller number of indicators (i.e., parcels) for the construct. It is a common technique used in structural model testing when a large number of variables load on a single latent construct (Graham et al., 2000). Parceling stabilizes parameter estimates and improves goodness of fit (Rogers and Schmitt, 2004) and is used in estimating structural models with non-normal variables and/or a relatively small sample size (West et al., 1995; Little et al., 2013).

In this study we parceled PA-E and PA-L. PA-E had five items, and all loaded on one single dimension in exploratory factor analysis (Cronbach’s α = 0.85). In this situation, items with the highest and lowest factor loadings were aggregated into the first parcel, followed by the aggregation of the items with the second highest and second lowest factor loadings, and lastly, the remaining item was the third parcel (Little et al., 2013).

PA-L had nine items: walking, jogging, and other physical activity during three periods (afterschool, Saturday, and Sunday). For each type of physical activity, the items of three periods had a good level of internal consistency, with the Cronbach’s α being 0.76, 0.85, and 0.73 for walking, jogging, and other physical activity, respectively. To maintain the conceptual meaning of the parcels, we adopted an internal consistency parceling approach and created three parcels by average the items of three time periods for each type of physical activity. *Parcel#1 – Walking*, included afterschool walking, Saturday walking, and Sunday walking. *Parcel#2 – Jogging*, included afterschool jogging, Saturday jogging, and Sunday jogging. *Parcel#3 – Other Physical Activity*, included afterschool other physical activity, Saturday other physical activity, and Sunday other physical activity.

#### Estimation of Structural Models

**FIGURE 1:**
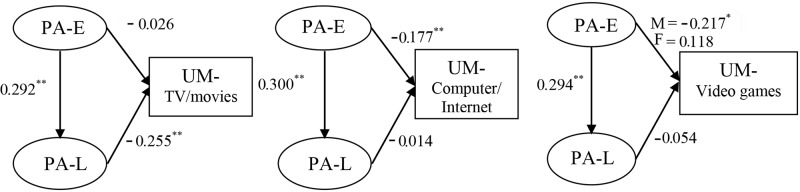
Standardized path coefficients in the structural models. ^∗^*p* < 0.05; ^∗∗^*p* < 0.01.

Multiple-group structural equation modeling (SEM) was used to examine the structural relationships between PA-E, PA-L, and UM for female and male adolescents. We built a separate structural model for each type of screen-based media (see **Figure 1**). Prior to that, multiple-group confirmatory factor analysis (CFA) was used to test the measurement invariance, which involved comparison of the model fit of two measurement models: (1) a configural invariance model in which the factor structure was fixed as the same across the two groups and then (2) a nested model (i.e., factor loading invariance model) where factor loadings were constrained to be equal across the two groups (MacCallum et al., 1994). The goodness of fit indices of the first model indicated how well the same factor structure fit the two groups, while the difference in goodness of fit between the second and the first model was used to assess factor loading invariance (i.e., measurement invariance).

After ensuring measurement invariance, the relationships between PA-E, PA-L, and UM (i.e., path coefficients) were examined between the two groups (Bollen, 1989). First, a baseline model was estimated in which all path coefficients were freely estimated for the two groups. Then a nested model was estimated in which all path coefficients were constrained to be equal across the two groups. The Chi-square difference between the two models was used to assess the overall path coefficients invariance. In situations where the Chi-square difference for the overall test was significant (*p* < 0.05) or marginally significant (*p* < 0.10), the equality constraint was put on each individual path coefficient to find out the one(s) that vary across groups. LISREL 9.20 was used to perform the Multiple-Group CFA and SEM. Full information maximum likelihood (FIML) approach was used to handle the missing data (4%) in model estimation. The sample size was considered sufficiently large given that it was more than 10 times of the number of estimated parameters in the study models (Kline, 2011).

## Results

### Descriptives

Overall male and female adolescents’ mean scores differed for all constructs (**Table 1**). On average, males had higher levels of PA-E (*M*_males_ = 4.08 vs. *M*_females_ = 3.76, *p* < 0.01) and PA-L (*M*_males_ = 4.00 vs. *M*_females_ = 3.36, *p* < 0.01). In terms of UM, females on average reported higher levels of TV/movie watching (*M*_males_ = 4.47 vs. *M*_females_ = 4.94, *p* < 0.05) and use of computer/Internet (*M*_males_ = 3.51 vs. *M*_females_ = 4.33, *p* < 0.01), while males on average spent more time on non-active video games (*M*_males_ = 3.72 vs. *M*_females_ = 2.04, *p* < 0.01).

**Table 1:** Descriptive statistics of the constructs.

	Gender	*N*	*M*	*SD*	*t*	*p*
PA-E	Male	146	4.08	0.939	2.954	0.003
	Female	134	3.76	0.898		
PA-L	Male	148	4.00	1.239	4.331	0.000
	Female	132	3.36	1.233		
UM-TV/movies	Male	155	4.47	1.945	-2.026	0.043
	Female	143	4.94	2.060		
UM-Computer/Internet	Male	153	3.51	2.193	-3.083	0.002
	Female	143	4.33	2.404		
UM- Video games	Male	156	3.72	2.106	7.732	0.000
	Female	144	2.04	1.643		

*PA-E, five-point scale; PA-L, seven-point scale; UM, eight-point scale.*

### Test of Measurement Invariance

For all three models, the measurement model achieved an acceptable fit when the same factorial structure was specified for males and females [TV/movies model: χ^2^(24) = 47.91, *p* < 0.01, RMSEA = 0.081, CFI = 0.968, NNFI = 0.943; computer/Internet model: χ^2^(24) = 42.01, *p* < 0.05, RMSEA = 0.070, CFI = 0.975, NNFI = 0.956; video games model: χ^2^(24) = 50.54, *p* < 0.01, RMSEA = 0.085, CFI = 0.964, NNFI = 0.936]. When the factor loadings were constrained to be equal between males and females, the change in model fit was non-significant for all three models [TV/movies model: Δχ^2^(4) = 3.88, *p* = 0.42; computer/Internet model: Δχ^2^(4) = 3.96, *p* = 0.41; video games model: Δχ^2^(4) = 3.73, *p* = 0.44]. These analyses suggested measurement invariance between males and females. For each model, all the factor loadings were highly significant and larger than 0.65 (*p* < 0.001; **Table 2**), indicating good convergent validity of the measurement (Cole, 1987). In terms of construct reliability, the score for PA-E and PA-L were above 0.75 for males and females in all three models. This indicated that overall the measurement models had good construct reliability (Hair et al., 2006). Finally, the average variance extracted for PA-E and PA-L exceeded their maximum squared between-construct correlations for both genders in all three models. This result indicated that the discriminant validity of the measurement was established (Hair et al., 2006).

**Table 2:** Standardized factor loadings in confirmatory factor analyses.

	TV/movies model	Computer/Internet model	Video games model
**Leisure time physical activity experience**			
PA-E1	0.918	0.915	0.924
PA-E2	0.802	0.806	0.796
PA-E3	0.702	0.701	0.700
**Leisure time physical activity levels**			
PA-L1	0.670	0.676	0.678
PA-L2	0.918	0.910	0.908
PA-L3	0.684	0.687	0.688

### Test of Path Coefficients Invariance

When the path coefficients were freely estimated for males and females, all three models (i.e., the baseline models) had an acceptable fit [TV/movies model: χ^2^(28) = 51.79, *p* < 0.01, RMSEA = 0.075, CFI = 0.968, NNFI = 0.952; computer/Internet model: χ^2^(28) = 45.97, *p* < 0.05, RMSEA = 0.065, CFI = 0.975, NNFI = 0.963; video games model: χ^2^(28) = 54.27, *p* < 0.01, RMSEA = 0.078, CFI = 0.964, NNFI = 0.946]. After all the path coefficients were constrained to be equal across the two groups, a marginally significant change in Chi-square was observed for the video games model [Δχ^2^(3) = 7.75, *p* = 0.051], while the model fit change was non-significant for the other two models [TV/movies model: Δχ^2^(3) = 2.96, *p* = 0.40; computer/Internet model: Δχ^2^(3) = 0.31, *p* = 0.96]. Subsequent analyses showed that males and females only significantly differed in the relationship between PA-E and use of non-active video games [Δχ^2^(1) = 5.99, *p* < 0.05]. Therefore, in the final estimation for the video games model, the PA-E – video games path was estimated freely for males and females, while the other path coefficients were fixed as the same between males and females.

**Table 3** and **Figure 1** showed the final results of model estimation. For the TV/movies model, PA-E had a significant positive effect on PA-L (β = 0.292, *p* < 0.01), and PA-L in turn had a significant negative effect on UM-TV/movies (β = -0.255, *p* < 0.01), indicating that the effect of PA-L on UM-TV/movies was mediated by PA-E (MacKinnon et al., 2002). This conclusion was also supported by the significant indirect effect. After accounting for PA-L, PA-E had a non-significant negative direct effect on UM-TV/movies (β = -0.026, *p* > 0.10). With respect to the computer/Internet model, PA-E had a significant positive effect on PA-L (β = 0.300, *p* < 0.01) and a significant direct negative effect on UM-computer/Internet (β = -0.177, *p* < 0.01). PA-L, however, had a non-significant negative effect on UM-Computer/Internet (β = -0.014, *p* > 0.10). Lastly for the video games model, PA-E had a significant positive effect on PA-L (β = 0.294, *p* < 0.01), while PA-L had a non-significant negative effect on UM-video games (β = -0.054, *p* > 0.10). PA-E had a significant direct negative effect on UM-video games among males (β = -0.217, *p* < 0.05), but a non-significant direct positive effect on UM-video games among females (β = 0.118, *p* > 0.10).

**Table 3:** Estimation of path coefficients, total effects, and indirect effects.

	Path coefficients		Total effect^†^	Indirect effect^†^
	Unstandardized	Standardized		
**TV/movies model^§^**				
PA-E → PA-L	0.323**	0.292**	0.323**	
PA-L → TV/movies	-0.530**	-0.255**	-0.530**	
PA-E → TV/movies	-0.059	-0.026	-0.230	-0.171**
**Computer/Internet model^§^**				
PA-E → PA-L	0.336**	0.300**	0.336**	
PA-L → Computer/Internet	-0.033	-0.014	-0.033	
PA-E → Computer/Internet	-0.468**	-0.177**	-0.479**	-0.011
**Video game model^§^**				
PA-E → PA-L	0.326**	0.294**	0.326**	
PA-L → Video games	-0.106	-0.054	-0.106	
PA-E → Video games (Male)	-0.471*	-0.217*	-0.506**	-0.035
PA-E → Video games (Female)	0.255	0.118	0.221	-0.035

*^§^TV/movies model: χ^2^(31) = 54.75, p < 0.01, RMSEA = 0.071, CFI = 0.968, NNFI = 0.956; Computer/Internet model: χ^2^(31) = 46.28, p < 0.05, RMSEA = 0.057, CFI = 0.979, NNFI = 0.971; Video games model: χ^2^(30) = 54.51, p < 0.01, RMSEA = 0.073, CFI = 0.967, NNFI = 0.953. ^†^Unstandardized total and indirect effects. ^∗^p < 0.05; ^∗∗^p < 0.01.*

## Discussion

Using a sample predominantly comprised of urban Latino and African American adolescents, we found that physical activity experience was positively associated with physical activity level, which was consistent with previous studies (Sallis et al., 1999; Dishman et al., 2005). After accounting for physical activity level, physical activity experience had a direct negative relationship with use of non-active video games for males and a direct negative effect on use of computer/Internet for both genders. Physical activity level did not have a direct relationship with the use of these two media. However, physical activity level had a direct negative relationship with use of TV/movies, while the relationship of physical activity experience to use of TV/movies was fully mediated by physical activity level.

These results support that physical activity experience may have a unique impact on use of certain screen-based media. In this study the unique impact of physical activity experience was reflected with the use of computer/Internet and non-active video games, but not the use of TV/movies. One possible reason is that these three types of media offer somewhat different types of experiences, and as a result, they are differentially substituted by physical activity experience. This is generally consistent with use and gratification theory, which suggests different media offer different experiences and that individuals deliberately choose media that will satisfy different needs (McQuail, 2010). For example, TV/movies normally provides passive entertainment, while use of computer/Internet and non-active video games usually requires more active engagement (Pine and Gilmore, 1999). Since physical activity also offers experiences that require active engagement, it is possible that physical activity experience is more effective in substituting experience offered by computer/Internet and non-active video games than the experience offered by TV/movies. In addition, compared to TV/movies, computer/Internet and video games may provide adolescents certain social (e.g., social networking) and achievement (e.g., accomplishing tasks in video/Internet games) experiences. These types of experiences are also likely to be gained from doing physical activities (Fox, 1999; Washington et al., 2001). To test this speculation on experience substitution, researchers may need to collect data on adolescents’ experience from using different screen-based media. In addition, although the physical activity measure in this study included different types of positive experiences (e.g., enjoyment, sense of accomplishment) obtained from physical activity, results of factor analysis showed that they all loaded into one dimension. It is possible that different types of positive experiences are highly correlated at early adolescence. In late adolescence, however, those experiences may emerge as distinct dimensions and may have somewhat different association with physical activity and screen-based media use.

The findings also suggest that the relationship between physical activity level and use of screen-based media is complicated and may vary for different types of media use. The non-significant relationship of physical activity level to use of computer/Internet and non-active video games does not necessarily mean that physical activity level does not affect adolescents’ use of these two media, but may indicate that physical activity level affects these media use through multiple mechanisms with opposite directions (Shrout and Bolger, 2002). On one hand, when adolescents participate in more physical activities, they may have less time available for screen-based media. On the other hand, doing more physical activity may make adolescents feel more justified for using screen-based media (i.e., licensing effect) or more difficult to resist the temptation of screen-based media (i.e., reduced self-regulation resource; Schmeichel and Baumeister, 2004). In addition, parents may feel more comfortable to let adolescents use screen-based media when the adolescents participate in physical activity. Therefore, future research should investigate these possible psychological and behavioral mechanisms.

The path coefficients in the structural models were generally similar between male and female adolescents, except for the relationship of physical activity experience to use of non-active video games. Physical activity experience had a significant negative relationship with use of non-active video games for males, but a non-significant positive relationship for females. This may suggest that males and females had different behaviors and experiences related to use of non-active video games. In addition to the fact that males played video games much more frequently than females (*M*_males_ = 3.72 vs. *M*_females_ = 2.04), the two gender groups may play different types of video games and therefore gain different types of experiences from video games.

This study has a few limitations. First, the data are cross-sectional. Although the data were useful in revealing associations between constructs, we were unable to test causal relationships. Therefore, future research should adopt experimental design and/or longitudinal data analysis. In addition, researchers may use qualitative approaches such as in-depth interviews to better understand the experiences adolescents obtain from leisure time physical activity and screen-based media, and improve measurement models based on the qualitative finding. Second, adolescents’ use of screen-based media may have changed since the data were collected approximately 7 years ago. For example, more children and adolescents now have the access to smart phones and tablets (Common Sense Media, 2013; Influence-Central, 2016), which makes it more convenient to watch TV/movies and play videogames. As a result, today’s adolescents may be more likely to resort to screen-based media in the absence of positive physical activity experience (i.e., a stronger relationship between physical activity experience and use of screen-based media). Therefore, researchers may use more current data to examine if some of the relationships in our model have changed. Third, this study only included positive physical activity experience. Future research should also examine the impact of negative physical activity experience, which may represent a separate dimension from positive physical activity experience.

## Conclusion

This study suggests that positive leisure time physical activity experience is associated with higher leisure time physical activity levels and has a negative association with use of non-active video games for male adolescents and use of computer/Internet for both male and female adolescents after controlling for physical activity levels. These finding suggest avenues for developing interventions to promote leisure time physical activity. For example, during an intervention or education sessions researchers or practitioners should consider promoting adolescents’ positive physical activity experience or adolescents’ competencies in creating positive physical activity experience, instead of solely focusing on increasing adolescents’ physical activity levels. Promoting positive physical activity experience may be particularly important to urban Latino/African American adolescents, many of whom are from families of low socioeconomic status. On one hand, it is more challenging for these adolescents to obtain positive physical activity experience due to limited resources and opportunities for physical activities (Dahmann et al., 2010). On the other hand, these adolescents tend to have lower self-regulation skills (Raver, 2012) and less monitoring and support from parents (Rideout et al., 2011). As a result, they may be more likely to be stuck with screen-based media when they do not obtain optimal experience from physical activity. In addition to highlighting the importance of leisure time physical activity experience, this study calls for future studies to refine relevant measurement and examine the determinants and developmental outcomes of physical activity experience.

## Author Contributions

HX, JS, and LC: substantial contributions to the conception or design of the work; or the acquisition, analysis, or interpretation of data for the work; drafting the work or revising it critically for important intellectual content; final approval of the version to be published; agreement to be accountable for all aspects of the work in ensuring that questions related to the accuracy or integrity of any part of the work are appropriately investigated and resolved.

## Conflict of Interest Statement

The authors declare that the research was conducted in the absence of any commercial or financial relationships that could be construed as a potential conflict of interest.
